# Valorization of Coffee Silverskin via Integrated Biorefinery for the Production of Bioactive Peptides and Xylooligosaccharides: Functional and Prebiotic Properties

**DOI:** 10.3390/foods14152745

**Published:** 2025-08-06

**Authors:** Thanongsak Chaiyaso, Kamon Yakul, Wilasinee Jirarat, Wanaporn Tapingkae, Noppol Leksawasdi, Pornchai Rachtanapun

**Affiliations:** 1 Division of Biotechnology, Faculty of Agro-Industry, Chiang Mai University, Chiang Mai 50100, Thailand; kamon.y@cmu.ac.th (K.Y.); jirarat4824@gmail.com (W.J.); 2 Center of Excellence in Agro Bio-Circular-Green Industry (Agro BCG), Faculty of Agro-Industry, Chiang Mai University, 155 Moo 2, Mae Hia, Muang, Chiang Mai 50100, Thailand; noppol.l@cmu.ac.th (N.L.); pornchai.r@cmu.ac.th (P.R.); 3 Department of Animal and Aquatic Sciences, Faculty of Agriculture, Chiang Mai University, Chiang Mai 50200, Thailand; wanaporn.t@cmu.ac.th; 4 Division of Food Engineering, Faculty of Agro-Industry, Chiang Mai University, Chiang Mai 50100, Thailand; 5 Division of Packaging Technology, Faculty of Agro-Industry, Chiang Mai University, Chiang Mai 50100, Thailand

**Keywords:** coffee silverskin (CS), bioactive peptides, xylooligosaccharides (XOS), antioxidant activity, ACE-inhibition, in vitro, prebiotic property

## Abstract

Coffee silverskin (CS), a by-product generated during coffee roasting, contains high levels of xylan hemicellulose and protein, making it a promising substrate for functional ingredient production. This study developed an integrated bioprocess to simultaneously produce bioactive peptides and xylooligosaccharides (CS-XOS) from CS. Conventional alkaline extraction (CAE) under optimized conditions (1.0 M NaOH, 90 °C, 30 min) yielded 80.64 mg of protein per gram of CS and rendered the solid residue suitable for XOS production. Enzymatic hydrolysis of the extracted protein using protease_SE5 generated low-molecular-weight peptides (0.302 ± 0.01 mg/mL), including FLGY, FYDTYY, and FDYGKY. These peptides were non-toxic, exhibited *in vitro* antioxidant activity (0–50%), and showed ACE-inhibitory activities of 60%, 26%, and 79%, and DPP-IV-inhibitory activities of 19%, 18%, and 0%, respectively. Concurrently, the alkaline-treated CS solid residue (ACSS) was hydrolyzed using recombinant endo-xylanase, yielding 52.5 ± 0.08 mg of CS-XOS per gram of ACSS. The CS-XOS exhibited prebiotic effects by enhancing the growth of probiotic lactic acid bacteria (μ_max_ 0.100–0.122 h^−1^), comparable to commercial XOS. This integrated bioprocess eliminates the need for separate processing lines, enhances resource efficiency, and provides a sustainable strategy for valorizing agro-industrial waste. The co-produced peptides and CS-XOS offer significant potential as functional food ingredients and nutraceuticals.

## 1. Introduction

The coffee production process generates a large amount of waste during the extraction of coffee beans. Only 40% of the coffee cherry is retained as beans, while the remaining 60% including pulp, mucilage, parchment, and silverskin are considered by-products [1,2]. According to the latest statistics from the International Coffee Organization (ICO), global coffee consumption in 2024 reached 177 million 60 kg bags, representing an increase of 2.2% compared to 2023. The rise in coffee consumption results in more coffee by-products that are challenging to manage. CS is the most abandoned product in the roasting process, with approximately 7.5 kg per 1 ton of roasted coffee beans [3]. Recent studies have investigated the valuable use and modification of CS for functional ingredients in the food, cosmetic, or medical industry, due to the high amount of fiber (38–45%) and protein (14–19%) [4,5,6].

In our previous study, the effect of various extraction methods on the quality of CS protein (CS-protein) to produce bioactive peptides was investigated. CS-protein was extracted using mild alkaline conditions, such as microwave-assisted alkaline extraction (MAE) and ultrasound-assisted alkaline extraction (UAE), and compared to conventional alkaline extraction (CAE). Although high-quality CS-protein suitable for bioactive peptide production was obtained from both MAE and CAE methods, the main drawback of these techniques lies in the difficulty of scaling up the processes for industrial application [7].

In addition, after protein extraction, the CS is still mainly composed of xylan hemicellulose, a potential substrate for XOS production via an enzymatic approach. These oligosaccharides have garnered attention for their possible health benefits, which include prebiotic effects, immune modulation, anti-inflammatory properties, enhanced mineral absorption, and improved glycemic control. Due to these potential health benefits, XOS have been applied as a potential functional ingredient in various food products, dietary supplements, and animal feeds [2,8,9]. Generally, pretreatment is necessary for the enzymatic production of XOS because it allows for better penetration of the enzyme into the hemicellulose structure. Alkaline pretreatment was found to be a low-cost and low-equipment-requirement process for breaking down the lignocellulose structure by removing the hydrogen bonds between lignin and cellulose, thereby increasing the accessibility of the lignocellulosic biomass structure [10]. Therefore, an alkaline solution is also a selective method widely employed for lignocellulosic biomass pretreatment in oligosaccharide production [11,12].

Recent studies have increasingly focused on valorizing CS into functional ingredients, such as prebiotics and bioactive peptides. For instance, Machado et al. [13] highlighted that CS contains high levels of dietary fiber and oligosaccharides with prebiotic and antioxidant properties. XOS derived from the enzymatic hydrolysis of CS xylan promoted the growth of *Lactobacillus casei* [14], while Machado et al. [15] demonstrated that ultrasonic-extracted CS enhanced the growth of *Lacticaseibacillus paracasei*, confirming its prebiotic potential. Protein extraction from CS was performed using MAE and UAE, with MAE demonstrating superior performance in protein recovery (43.5%) and quality retention [5]. Additionally, Ramírez et al. [16] reported that fermentation of protein in spent coffee grounds (SCG) using *Bacillus clausii* enhanced the soluble protein content and generated bioactive peptides exhibiting antioxidant and ACE-inhibitory activities. These recent developments also reflect the growing interest in utilizing CS as an ingredient in food products, particularly due to its fiber, prebiotic, and protein content, which supports its application in health-oriented and sustainable food innovations. Despite growing interest in CS valorization, few studies have addressed the simultaneous enhancement of protein and xylan fractions for the co-production of bioactive peptides and XOS.

Hence, this study investigates the effects of alkaline pretreatment processes on enhancing the quality of CS-protein for bioactive peptide production, while also serving as an effective pretreatment for CS xylan to facilitate the enzymatic production of CS-XOS. The overarching goal is to optimize the integrated bioprocess for the co-production of bioactive peptides and CS-XOS. Their potential applications as functional food ingredients and prebiotic properties are also examined. This study highlights a novel approach to valorizing CS by converting them into high-value bioactive compounds, thereby supporting sustainable waste utilization. The proposed strategy aligns with current directions in circular bioeconomy and zero-waste goals, offering distinct improvements over our previous work regarding process integration and application.

## 2. Materials and Methods

### 2.1. Materials and Reagents

Arabica CS was supported by the Department of Animal and Aquatic Sciences, Faculty of Agriculture, Chiang Mai University, Thailand. The dried CS was milled to a particle size of 100 mesh and kept in sealed plastic bags at 4 °C in the dark until use. The reagents for antioxidant activity analysis, including 2,2-diphenyl-1-picrylhydrazyl (DPPH), 2,2′-azinobis-(3-ethylbenzothiazoline-6-sulfonic acid (ABTS), and standard chemicals for HPLC analysis, were purchased from Sigma–Aldrich, (St. Louis, MO, USA). Other chemicals were analytical grade reagents. For the cytotoxicity investigation, murine macrophage RAW 264.7 cells (TIB–71TM) were purchased from the American Type Culture Collection (ATCC) (Manassas, VA, USA). Lipopolysaccharides (LPS) and Griess reagent were obtained from Sigma–Aldrich (St. Louis, MO, USA). Dulbecco’s Modified Eagle Medium (DMEM), fetal bovine serum (FBS), and penicillin–streptomycin were obtained from Thermo Fisher Scientific Inc. (Grand Island, NY, USA).

### 2.2. Pretreatment of CS and Protein Extraction

The hydrothermal (HT) process treated the CS as described by Jirarat et al., 2024 [7]. The 10% (*w*/*v*) of CS was mixed with distilled water and autoclaved at 125 °C for 25 min to extract phenolic compounds and remove undesirable materials. The HT-treated CS solid residue was then dried in a hot air oven at 60 °C for 12–18 h until a constant weight was achieved. Subsequently, the HT-treated CS (10.0% *w*/*v*) was suspended in 100 mL of NaOH solution in a 250 mL Duran bottle and subjected to protein extraction using either the MAE, UAE, or CAE method according to Jirarat et al. 2024 [7]. Briefly, 0.2 M NaOH was used for the MAE and UAE. The MAE was performed at an electric power of 90 W for 2, 5, and 10 min. The UAE was conducted at 20 kHz with an amplitude of 80% for 10, 25, and 40 min, respectively. CS was treated with 0.2–1.0 M NaOH solution at 90 °C during the CAE for 30 min. The mixture was cooled and centrifuged at 6000 rpm (4430× *g*) for 15 min. The supernatant was then recovered and subjected to protein precipitation by adjusting the pH to 3.5 using 6 N HCl, followed by storage at 4 °C overnight. The resulting precipitate was collected by centrifugation, as previously described, and subsequently freeze-dried using a Labconco FreezeZone 6 system (FreezeZone 6, Labconco Corporation, Kansas City, MO, USA). Meanwhile, the ACSS was thoroughly washed with distilled water until reaching a neutral pH (6–7), dried at 60 °C for 12 h, and subsequently used as a substrate for the enzymatic production of CS-XOS.

### 2.3. Microorganism and Enzyme Production

#### 2.3.1. Alkaline Protease (Protease_SE5) Production

The thermostable alkaline protease (protease_SE5) was produced in the production medium, which was composed of 1.0 g/L K_2_HPO_4_, 0.5 g/L NaCl, 0.1 g/L CaCl_2_·2H_2_O, 0.1 g/L MgSO_4_·7H_2_O, 0.2 g/L Tween 80, and 10 g/L urea. The starter culture of *Bacillus halodurans* SE5 (MH299852) was cultivated in the production medium at pH 9.7 and incubated at 45 °C, 200 rpm for 60 h [17]. The production medium was collected after 60 h by centrifugation at 6000 rpm (4430× *g*), 4 °C for 15 min. Subsequently, the supernatant was evaporated and dialyzed against 10 mM carbonate–sodium carbonate buffer pH 9.5. Protease_SE5, exhibiting an activity of 17200 U/mL and a specific activity of 1200 U/mg protein, was used for the enzymatic production of bioactive peptides from CS-protein.

#### 2.3.2. Endo-Xylanase Production

The recombinant endo-xylanase was produced in buffered minimal methanol-complex (BMMY) medium, which consisted of 10.0 g/L yeast extract, 20.0 g/L peptone, and 13.4 g/L of yeast nitrogen base. The starter culture of the recombinant *Pichia pastoris* GS115 was cultivated in BMMY medium pH 6.5 and incubated at 30 °C, 200 rpm for 5 days [18]. The *xylanase_1948* gene expression was induced by adding 4 × 10^−5^% (*w*/*v*) biotin, and 0.5% (*v*/*v*) methanol every 24 h. The crude enzyme was then harvested by centrifugation at 6000 rpm (4430× *g*), 4 °C, for 10 min. A 30 kDa molecular weight cut-off (MWCO) regenerated cellulose membrane (MilliporeSigma, Burlington, MA, USA) was used for the concentration and purification of recombinant endo-xylanase. The partially purified recombinant endo-xylanase, exhibiting an activity of 1650 U/mL and a specific activity of 170 U/mg protein, was used for the enzymatic production of XOS.

#### 2.3.3. Probiotics Preparation

Three probiotic lactic acid bacteria (LAB), *Lacticaseibacillus casei* TISTR1463, *Lactobacillus delbrueckii* subsp. *lactis* TISTR1464, and *Lactiplantibacillus plantarum* TISTR1465 were obtained by the Thailand Institute of Scientific and Technological Research (TISTR). For inoculum preparation, the strains were grown in de Man, Rogosa, and Sharpe (MRS) broth at 30 °C for 24 h until reaching an optical density (OD_600_) of 1.0. Cultures were then centrifuged at 6000 rpm (4430× *g*) at 4 °C for 10 min (Z236K, Hermle Labortechnik GmbH, Wehingen, Germany). The resulting cell pellets were washed twice and resuspended in sterile 0.85% (*w*/*v*) NaCl for prebiotic activity assays with XOS [19].

### 2.4. Effects of MAE, UAE, and CAE Methods on CS-XOS Production

The reaction was prepared by mixing 10% of ACSS (100 mL) obtained from the MAE, UAE, and CAE methods in 10 mM of phosphate buffer (KP-buffer) pH 6.5 in a 250 mL Duran bottle, followed by autoclaving at 121 °C for 15 min. The recombinant endo-xylanase (150 U/g CS) was added to the sterilized reaction and incubated at 50 °C, 100 rpm for 12 h. After incubation, the reaction was centrifuged at 6000 rpm (4430× *g*), 4 °C for 20 min to collect the supernatant [19]. The CS-XOS was evaporated and then mixed with an anion-exchange resin (diethylaminoethyl, DEAE resin) in a ratio of 1 g of DEAE per 10 mL of concentrated CS-XOS. The mixture was incubated at 4 °C for 30 min, followed by filtration using Whatman No.1 filter paper (GE Healthcare Life Sciences, Chicago, IL, USA), and the filtrate was then subjected to freeze-drying [19].

The scale-up of CS-XOS production was also investigated. The ACSS selected from the optimized process was mixed with 10 mM of potassium phosphate buffer pH 6.5 in a 5.0 L stirred-tank bioreactor (MDFT-N-5L, BE Marubishi, Pathum Thani, Thailand) with an agitation rate of 200 rpm. The recombinant xylanase (150 U/g ACSS) was then added to the sterilized reaction and incubated at 50 °C for 12 h. The downstream process of CS-XOS was carried out as previously described. Subsequently, the concentration of XOS and their prebiotic properties were evaluated according to the methods described by [19].

### 2.5. Enzymatic Production of Bioactive Peptide

#### 2.5.1. Enzymatic Production of Bioactive Peptide from CS-Protein

The freeze-dried CS-protein powder from the appropriate alkaline extraction process was used as a substrate for bioactive peptide production. Briefly, the enzymatic reaction was prepared by dissolving CS-protein powder into carbonate-sodium carbonate buffer pH 9.5 at a 5 mg/mL concentration. The protease_SE5 was then added to the reaction (200,000 U/g protein), and the reaction was incubated at 50 °C for 12 h to achieve complete protein hydrolysis [7].

#### 2.5.2. CS-Peptide Fractionation, Identification, and Synthesis

Peptide fractionation and identification were performed as described by Yakul et al. [17], with slight modifications. Briefly, low-molecular-weight (MW) peptides from the CS-protein hydrolysate were separated using an ultrafiltration (UF) centrifuge tube equipped with a 3 kDa molecular weight cut-off (MWCO) polyether sulfone (PES) membrane (MilliporeSigma, Burlington, MA, USA). The permeate containing peptides smaller than 3 kDa was further fractionated using a Sephadex G-25 gel filtration column (1.5 × 75 cm, column volume 125 mL). Elution was carried out with deionized water at a constant flow rate of 26 mL/h, and 2.5 mL fractions were collected per tube. The collected fractions were analyzed for peptide concentration, ABTS activity, and protein content. Fractions showing desirable characteristics were then pooled based on their absorbance at 280 nm (A_280_) and subsequently lyophilized.

The lyophilized peptide samples were submitted for de novo sequencing using liquid chromatography coupled with tandem mass spectrometry (LC-MS/MS) on a quadrupole time-of-flight (QTOF) system at the Salaya Central Instrument Facility, Mahidol University, Thailand. The MS/MS data were processed and analyzed using the PEAKS Studio Xpro software platform (Bioinformatics Solutions Inc., Waterloo, ON, Canada) with de novo peptide sequencing for peptide identification. The bioactivities of the identified peptides were predicted using BIOPREP, ToxinPred, and PeptideRanker. Peptides with a local confidence score ≥ 80% and an average local confidence (ALC) score > 90% were selected and subsequently synthesized as custom peptides with ≥ 95% purity by GenScript Ltd. (Piscataway, NJ, USA). Peptides with lower ALC scores were excluded from further analysis.

### 2.6. Analytical Method

#### 2.6.1. Chemical Composition of CS

The CS was milled and oven-dried at 60 °C for 12 h. The moisture content was determined using a moisture analyzer (MX-50, A&D, Toshima-Ku, Tokyo, Japan). The nitrogen content was measured by high-temperature combustion using a nitrogen analyzer (FP-528, LECO, St. Joseph, MI, USA), and the crude protein content was calculated using a conversion factor of 6.25. The crude lipid content was determined via Soxhlet extraction using petroleum ether as the solvent in a solvent extractor (SER 148 Series, VELP Scientific, Deer Park, NY, USA). The ash content was analyzed using a muffle furnace (Carbolite Gero, Hope Valley, UK). All proximate analyses, including moisture, protein, fat, and ash contents, were conducted according to the AOAC Official Methods [20].

#### 2.6.2. Analysis of Biological Activities and Cytotoxicity of Bioactive Peptide

##### Antioxidant Activity

Antioxidant activity analysis was performed in the dark. DPPH, ABTS radical scavenging activity, and the ferric-reducing antioxidant power (FRAP) assay were determined following the method described by Jirarat et al. (2024) [7]. Concisely, samples were diluted using distilled water to the desired concentration. For the DPPH assay, a 500 μL sample was mixed with 500 μL of ethanolic DPPH solution (80 mg/L) for 60 min before measuring the absorbance at 517 nm. The ABTS radical (ABTS+) solution was prepared by incubating the mixture of 2.45 mM potassium persulfate solution and 7 mM ABTS solution (1:1, *v*/*v*) for 12–16 h. The deionized water was used for diluting the ABTS^+^ solution to 0.70 ± 0.02 at an absorbance of 743 nm. After that, the ABTS assay was performed by incubating a 50 μL sample with 850 μL of prepared ABTS^+^ solution for 30 min, and the radical scavenging activity was determined by measuring the absorbance at 734 nm. Furthermore, the FRAP assay was performed by preparing the FRAP reagent, which consisted of 300 mM acetate buffer (pH 3.6), 20 mM FeCl_3_·6H_2_O, and 10 mM TPTZ in 40 mM HCl, mixed in a 10:1:1 volume ratio. Then, 100 μL of the sample was mixed with 900 μL the FRAP reagent and incubated for 30 min. Absorbance of the reaction mixture was measured at 595 nm using a spectrophotometer (Genesys™ 10S, Thermo Fisher Scientific, Waltham, MA, USA). The antioxidant capacity in all assays was expressed as micromoles of Trolox equivalents per liter (μmol TE/L) based on a Trolox standard curve.

##### Total Phenolic Content (TPC)

TPC was determined using the Folin–Ciocalteu colorimetric method with slight modifications [7]. Briefly, 300 μL of the sample was mixed with 400 μL of 0.2 M Folin–Ciocalteu reagent and incubated at room temperature for 5 min. Subsequently, 500 μL of 0.7 M sodium carbonate (Na_2_CO_3_) solution was added, and the mixture was incubated for 2 h. The absorbance was measured at 760 nm using a spectrophotometer. TPC was expressed as micromoles of gallic acid equivalents per liter (μmol GAE/L), based on a standard curve prepared with gallic acid.

##### Angiotensin-I-Converting Enzyme (ACE) Inhibition Activity

The ACE inhibitory activity of synthetic peptides was determined using a modified method described by Yakul et al. (2023) [21]. Briefly, 25 μL of the sample was pre-incubated with 25 μL of ACE solution (100 mU/mL) in a 1.5 mL microcentrifuge tube at 37 °C for 10 min. A control reaction was prepared by mixing ACE solution with 100 mM glycine–NaOH buffer (pH 9.5) containing 5 mM CaCl_2_. Subsequently, 75 μL of 4.15 mM hippuryl-histidyl-leucine (HHL) in 50 mM of sodium borate buffer (pH 8.3) containing 500 mM of NaCl was added to initiate the reaction. The mixture was incubated at 37 °C for 30 min, and the reaction was terminated by adding 125 μL of 1 M HCl. Then, 250 μL of pyridine and 125 μL of benzene sulfonyl chloride were added to the mixture to develop the colorimetric product. The absorbance was measured at 410 nm, and the ACE inhibitory activity (%) was calculated from the difference in absorbance between the control and the samples.

##### Dipeptidyl Peptidase-IV (DPP-IV) Inhibition Activity

The DPP-IV inhibitory activity was determined using a modified method based on Lacroix and Li-Chan [22]. Briefly, the synthetic peptide was diluted in 100 mM Tris-HCl buffer (pH 8.0) to a final concentration of 5000 μM. The reaction mixture was prepared in a 1.5 mL microcentrifuge tube by combining 75 μL of Gly-Pro-*p*-nitroanilide (1.59 mM), 50 μL of Tris-HCl buffer (pH 8.0), and 25 μL of the peptide sample. The mixture was pre-incubated at 37 °C for 10 min. Following pre-incubation, 50 μL of DPP-IV enzyme solution (0.01 U/mL) was added, and the reaction was incubated at 37 °C for 60 min. The reaction was terminated by adding 300 μL of 99.8% ethanol. The absorbance of the reaction mixture was measured at 385 nm, and the DPP-IV inhibitory activity was calculated and expressed as a percentage of inhibition.

##### Cytotoxicity Analysis

The cytotoxicity of synthetic bioactive peptides was evaluated using the sulforhodamine B (SRB) colorimetric assay, as described by Vichai and Kirtikara [23]. Briefly, RAW 264.7 murine macrophage cells were seeded at a 2 × 10^4^ cells/well density in 96-well plates and incubated for 24 h. The cells were then treated with various concentrations of peptides (0–200 μg/mL) for an additional 24 h. After treatment, the cells were fixed with cold trichloroacetic acid (TCA) and stained with 0.4% (*w*/*v*) SRB in 1% (*v*/*v*) acetic acid. Unbound dye was removed by washing, and protein-bound SRB was solubilized using 10 μM Tris base solution. Absorbance was measured at 520 nm using a microplate reader, and cell viability was calculated as a percentage relative to untreated control cells. Non-cytotoxic concentrations of the peptides were subsequently used for further investigation of their anti-inflammatory effects on LPS-induced nitric oxide (NO) production in RAW 264.7 cells.

##### Anti-Inflammatory Activity

The anti-inflammatory activity of synthetic peptides was evaluated based on the inhibition of nitric oxide (NO) production in RAW 264.7 cells, following the method described by Singai et al. [24]. Briefly, RAW 264.7 cells were seeded in 96-well plates at a density of 2 × 10^4^ cells/well and incubated for 24 h. The cells were then treated with various concentrations of synthetic peptides (0–200 μg/mL) for 2 h, followed by stimulation with or without 1 μg/mL of LPS. After 24 h of incubation, the culture supernatant was collected and mixed with Griess reagent to determine NO production. The absorbance was measured at 550 nm, and the NO concentration was calculated using a standard curve generated from sodium nitrite solutions.

#### 2.6.3. XOS, Sugars, and Lactic Acid Analysis

The utilization of CS-XOS and the production of lactic acid by LAB were quantified using high-performance liquid chromatography (HPLC), as described by Boonchuay et al. [19]. Analysis was carried out on an Aminex HPX-87H column maintained at 40 °C, using 5.0 mM H_2_SO_4_ as the mobile phase at a flow rate of 0.45 mL/min. The compounds were detected with a refractive index (RI) detector over a 30-min run. Concentrations were determined by calibration against respective XOS and lactic acid standards.

#### 2.6.4. Prebiotic Property of CS-XOS

The prebiotic potential of CS-XOS was evaluated by assessing its ability to support the growth of probiotic LAB strains. MRS medium supplemented with concentrated CS-XOS (30 g/L) as the sole carbon source was used for cultivation, while MRS containing 30 g/L glucose served as the control. Three LAB strains were individually inoculated into MRS media supplemented with CS-XOS or commercial XOS. Cultures were incubated at 30 °C, and samples were collected at 0, 6, 12, 18, 24, 36, and 48 h of cultivation. Bacterial growth was monitored by determining viable cell counts (CFU/mL) and maximum specific growth rate (μ_max_). Simultaneously, CS-XOS consumption and lactic acid production were analyzed using HPLC, following the method of Boonchuay et al. [19].

#### 2.6.5. Statistical Data Analysis

All experiments were performed in triplicate (*n* = 3). The data’s normality was confirmed before ANOVA, and all factors were considered fixed effects. The data were analyzed for statistical significance using one-way analysis of variance (ANOVA) followed by Duncan’s multiple range test (*p* ≤ 0.05). The statistical software SPSS v.17.0 (SPSS Inc., Chicago, IL, USA) was used to analyze the experimental data.

## 3. Results

### 3.1. Effects of Extraction Methods on CS-XOS Production

**Figure 1:**
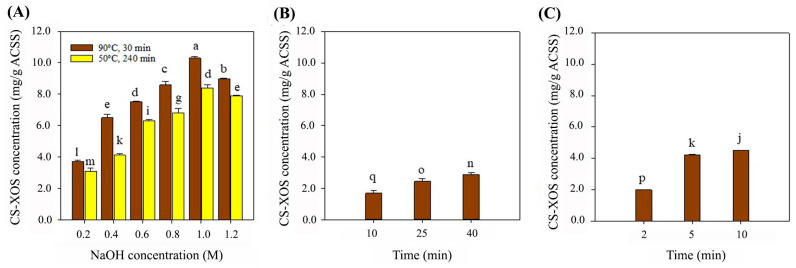
CS-XOS concentrations were determined from ACSS obtained through different alkaline extraction methods (CAE, UAE, and MAE). In CAE, CS (10%, *w*/*v*) was extracted using 0.2, 0.4, 0.6, 0.8, 1.0, and 1.2 M NaOH at 50 °C for 240 min (yellow bars), and at 90 °C for 30 min (brown bars) (**A**). In UAE, CS (10%, *w*/*v*) was extracted using 0.2 M NaOH for 10, 25, and 40 min (**B**). In MAE, CS (10%, *w*/*v*) was treated with 0.2 M NaOH under microwave irradiation at 90 W for 2, 5, and 10 min (**C**). CS-XOS hydrolysis was performed using recombinant endo-xylanase (150 U/g ACSS) at pH 6.5 and 50 °C for 12 h. CS-XOS hydrolysis was performed using recombinant endo-xylanase (150 U/g ACSS) at pH 6.5, 50 °C for 12 h. The experiments are performed in triplicate (*n* = 3). Different letters indicate significant differences at *p* ≤ 0.05 according to Duncan’s multiple range test analysis.

CS-XOS were enzymatically produced using ACSS, which were obtained from CS-protein extraction by the MAE, UAE, and CAE methods. These substrates were hydrolyzed using recombinant endo-xylanase. As shown in Figure 1A, the highest XOS yield (10.30 mg/g ACSS) was obtained from the CAE method under the condition of 1.0 M NaOH at 90 °C for 30 min. However, increasing the alkaline concentration to 1.2 M NaOH under milder temperature or prolonged extraction conditions significantly reduced XOS production. Specifically, XOS concentrations dropped to 7.90 ± 0.04 mg/g ACSS under 1.2 M NaOH at 50 °C for 240 min, and to 8.97 ± 0.05 mg/g ACSS under 1.2 M NaOH at 90 °C for 30 min. Conversely, the lowest XOS yields of 3.10 ± 0.20 and 3.70 ± 0.10 mg/g ACSS were observed under low alkaline conditions (0.2 M NaOH at 50 °C for 240 min and 90 °C for 30 min, respectively). Figure 1B,C show the XOS concentrations from ACSS derived via the UAE and MAE methods. For UAE, the XOS yields at 10, 25, and 40 min of extraction were 1.71 ± 0.07, 2.45 ± 0.02, and 2.88 ± 0.01 mg/g ACSS, respectively. In the case of MAE, treated ACSS at 2, 5, and 10 min resulted in XOS concentrations of 0.99 ± 0.01, 4.20 ± 0.04, and 4.51 ± 0.01 mg/g ACSS, respectively. Therefore, the CAE condition using 1.0 M NaOH at 90 °C for 30 min was the optimal treatment for maximizing CS-XOS production.

### 3.2. Bioreactor-Scale Integrated Process of Bioactive Peptide and CS-XOS Production

The CS-protein obtained from the CAE (1.0 M NaOH, 90 °C, 30 min) was hydrolyzed using protease_SE5. The hydrolysis reactions were conducted in a 250 mL Duran bottle and a 5.0 L stirred-tank bioreactor. The protein concentration, peptide concentration, and antioxidant activity were subsequently analyzed. As shown in Table 1, the peptide concentration and antioxidant activity of CS-protein hydrolysate were 0.323 ± 0.00 mg/mL and 1501 ± 21 μmol TE/L in the flask-scale experiment, and 0.302 ± 0.01 mg/mL and 1511 ± 58 μmol TE/L in the bioreactor-scale experiment, respectively. These results indicate no significant differences in peptide concentration and antioxidant activity between the two production scales. Therefore, CS-protein extraction was performed using the CAE in a stirred-tank bioreactor with 3.0 L of working volume.

### 3.3. CS-Peptides Fractionation

**Figure 2:**
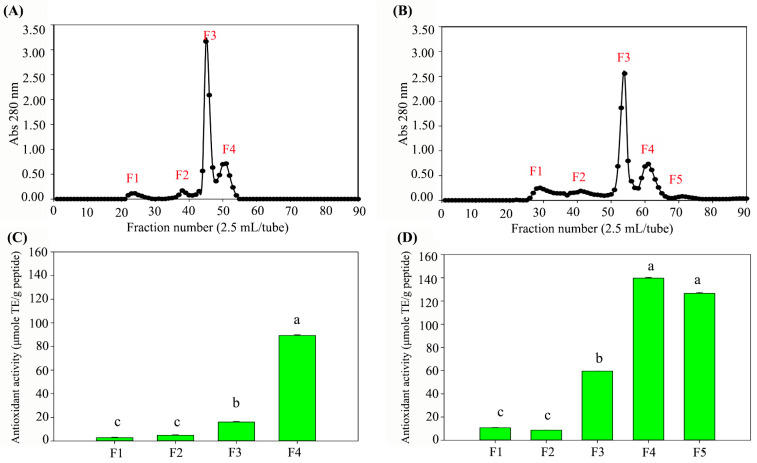
Fractionation and antioxidant activity of CS-protein and CS-protein hydrolysate. CS-protein (**A**) and CS-protein hydrolysate (**B**); antioxidant activity (ABTS assay) of the fractionated CS-protein (**C**) and CS-protein hydrolysate (**D**). CS-protein hydrolysate was prepared by dissolving CS-protein powder (5 mg/mL) in carbonate–sodium carbonate buffer (pH 9.5), followed by enzymatic hydrolysis with protease_SE5 (200,000 U/g protein, 50 °C, 12 h). Low-molecular-weight peptides from CS-protein and its hydrolysate were separated using a 3 kDa MWCO ultrafiltration tube. The permeates (<3 kDa) were further fractionated using a Sephadex G-25 gel filtration column (1.5 × 75 cm, 125 mL), eluted with deionized water at a flow rate of 26 mL/h. Fractions (2.5 mL) were collected and analyzed for absorbance at 280 nm and ABTS radical scavenging activity. The samples were fractionated, analyzed in triplicate, and examined through Duncan’s multiple range test. The different letters (a–c) indicated the significant data difference at *p* ≤ 0.05.

UF and subsequent gel filtration analysis revealed that the CS-protein sample exhibited four peptide peaks, while the hydrolysate demonstrated five peaks (Figure 2A,B). The CS-protein hydrolysate showed higher peak intensities (A_280_) than the non-hydrolyzed CS-protein, indicating a higher concentration of released peptides. As a result, the antioxidant activity of the hydrolyzed peptide fractions was dramatically higher than that of the non-hydrolyzed fractions (Figure 2C,D). Based on their high antioxidant activity, fractions 3 (F3), 4 (F4), and 5 (F5) from both the CS-protein and CS-protein hydrolysate samples were selected for further characterization using LC-MS/MS.

### 3.4. CS-Peptide Identification and Predicted Biological Activity

LC-MS/MS analysis demonstrated variations in the peptide profiles of the non-hydrolyzed CS-protein compared to the hydrolysate produced using protease_SE5. Several unique peptide sequences were identified only in the hydrolyzed sample, indicating that enzymatic hydrolysis significantly changed the peptide composition (see Supplementary Data). Among the identified peptides in the hydrolyzed CS-protein samples, three sequences were highlighted for their potential bioactivity, namely phenylalanine-leucine-glycine-tyrosine (Phe-Leu-Gly-Tyr, FLGY) obtained from F3, phenylalanine-tyrosine-aspartic acid-threonine-tyrosine-tyrosine (Phe-Tyr-Asp-Thr-Tyr-Try, FYDTYY), and phenylalanine-aspartic acid-tyrosine-glycine-lysine-tyrosine (Phe-Asp-Tyr-Gly-Lys-Tyr, FDYGKY) obtained from F4.

The selected peptides predicted to have bioactivity were commercially synthesized (GenScript, Piscataway, NJ, USA) and subjected to *in vitro* evaluation. ACE inhibitory activity was assessed at a peptide concentration of 500 μM, while DPP-IV inhibitory activity and antioxidant activity (ABTS radical scavenging assay) were evaluated at a concentration of 5000 μM. The results demonstrate that FLGY, FYDTYY, and FDYGKY exhibited notable bioactivities. Specifically, FLGY showed 60% ACE inhibition, 19% DPP-IV inhibition, and 48% ABTS radical scavenging activity. FYDTYY exhibited 26% ACE inhibition and 18% DPP-IV inhibition. Notably, FDYGKY displayed the highest ACE inhibitory activity among the tested peptides, with 79% inhibition (Table 2). In addition, the cytotoxicity and anti-inflammatory activities of the three synthetic peptides were further evaluated using murine macrophage RAW 264.7 cells.

**Table 2:** Analysis of bioactivity of peptides (ALC score > 90%), derived from CS-protein hydrolysate.

No.	Peptide Sequence	BIOPEP *			*In Vitro* Analysis		
		ACE Inhibitory Activity	DPP-IV Inhibitory Activity	Antioxidant Activity	ACE Inhibition ** (%)	DPP-IV Inhibition *** (%)	Antioxidant Activity ** (%)
1	FLGY	🗸	🗸	-	60 ± 1 ^b^	19 ± 1 ^a^	48 ± 1 ^a^
2	FYDTYY	🗸	🗸	-	26 ± 7 ^c^	18 ± 3 ^a^	N.D.
3	FDYGKY	🗸	-	-	79 ± 7 ^a^	N.D.	50 ± 1 ^a^

Note: The experiments are performed in triplicate (*n* = 3). The results are reported as mean ± SD. Different letters (a–c) within columns are significantly different at *p* ≤ 0.05 according to analysis by Duncan’s multiple range test. * Prediction of the biological activity by BIOPEP, available at http://www.uwm.edu.pl/biochemia/index.php/pl/biopep (accessed on 20 January 2025). ** ACE inhibition activity and antioxidant activity of peptides were evaluated at a peptide concentration of 500 μM. *** DPP-IV inhibitory activity of the peptides was assessed at a peptide concentration of 5000 μM.

### 3.5. Cytotoxicity of Synthetic Peptides in Murine Macrophage Cells

**Figure 3:**
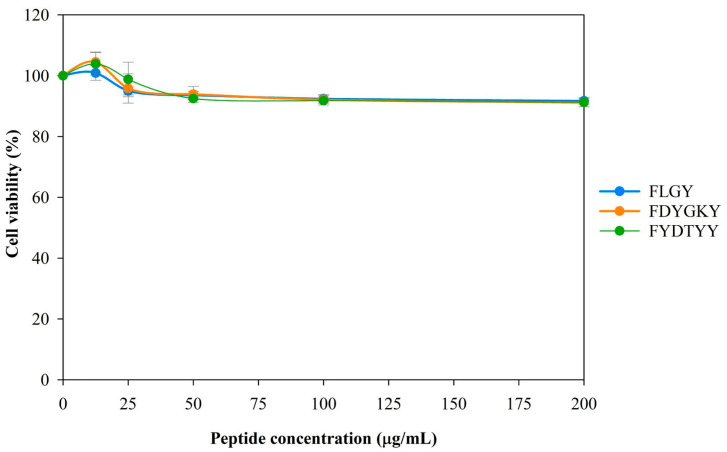
Cell viability of synthetic peptides in RAW 264.7 cells. RAW 264.7 cells were treated with different concentrations of various peptides (0–200 μg/mL) for 24 h. Cell viability was determined by the SRB assay. The data are shown as the mean ± SD of three independent experiments.

To investigate the potential application of three peptides, their cytotoxicity was evaluated in murine macrophage RAW 264.7 cells. The cells were treated with increasing concentrations (0–200 µg/mL) of each peptide for 24 h. Based on the SRB assay, all peptides demonstrated no cytotoxic effects at 200 µg/mL concentrations, with cell viability exceeding 90% (Figure 3).

### 3.6. Anti-Inflammatory Activity of Synthetic Peptides in Murine Macrophage Cells

**Figure 4:**
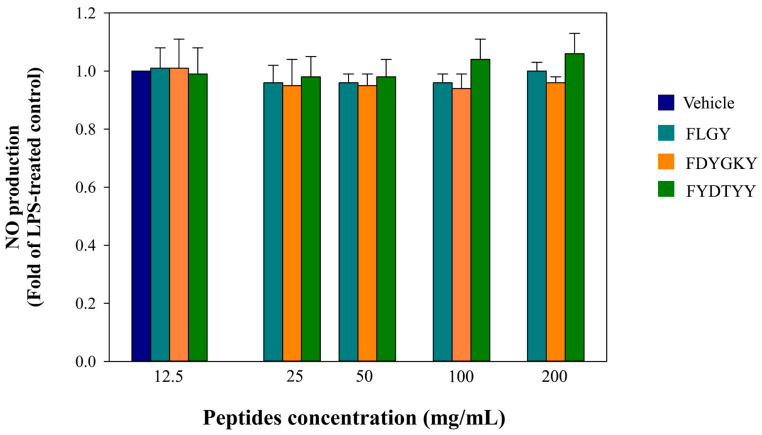
Effects of synthetic peptides on LPS-induced NO production in RAW 264.7 cells. RAW 264.7 cells were treated with various peptides (0–200 μg/mL). After treatment (2 h), the cells were treated with 1 μg/mL of LPS and then incubated for 24 h. The supernatant was collected to determine the NO production by Griess reagent. The data are shown as the mean ± SD of three independent experiments.

Several anti-inflammatory peptides have been identified through the proteolytic digestion of edible proteins. Much of the current knowledge regarding their anti-inflammatory properties has been obtained from *in vitro* studies [25]. In the present study, the potential anti-inflammatory effects of three peptides were evaluated in LPS-stimulated RAW 264.7 macrophages. Nitric oxide (NO) is a key pro-inflammatory mediator that contributes to the inflammatory response when produced in excess under pathological conditions [26]. The results indicate that none of the three peptides significantly inhibited LPS-induced NO production (Figure 4). However, without LPS stimulation, these peptides did not induce NO production, suggesting that they do not exhibit intrinsic pro-inflammatory activity.

### 3.7. Bioreactor-Scale Production of CS-XOS

The XOS concentration, antioxidant activity, and TPC of CS-XOS produced under the flask and bioreactor scales are summarized in Table 3. Antioxidant activity was determined using ABTS, DPPH, and FRAP assays. Additionally, the TPC of CS-XOS was also determined. The results indicate no significant differences in antioxidant activity or TPC of CS-XOS between the flask and bioreactor scales. However, the XOS yield was notably improved when the process was scaled up to the 5.0 L stirred-tank bioreactor. Specifically, flask-scale production yielded 20.30 mg XOS per gram of ACSS, whereas bioreactor-scale production yielded 52.50 mg XOS.

### 3.8. Prebiotic Property of CS-XOS

To evaluate the prebiotic potential of CS-XOS, the reduction in XOS content, the increase in viable cell counts, and the accumulation of lactic acid were analyzed (Table 4). In addition, the kinetic parameters of probiotic LAB cultivated in MRS medium supplemented with either commercial XOS or CS-XOS were calculated. As shown in Table 4, *Lacticaseibacillus casei* TISTR1463 exhibited similar utilization patterns for both commercial XOS and CS-XOS. Commercial XOS and CS-XOS concentrations in the MRS medium during fermentation decreased by 5.59 ± 0.05 mg/mL and 5.16 ± 0.17 mg/mL, respectively. In contrast, *Lactobacillus delbrueckii* subsp. *lactis* TISTR1464 consumed more commercial XOS than CS-XOS. While *Lactiplantibacillus plantarum* TISTR1465 consumed comparable amounts of commercial XOS (4.22 ± 0.14 mg/mL) and CS-XOS (4.23 ± 0.17 mg/mL).

Regarding the μ_max_ values and lactic acid production by strain TISTR1463, supplementation with CS-XOS in MRS medium enhanced μ_max_ to 0.021 ± 0.001 h^−1^ and lactic acid concentration to 8.49 ± 0.45 mg/mL. Similarly, commercial XOS supplementation increased μ_max_ and lactic acid production to 0.103 ± 0.001 h^−1^ and 9.19 ± 0.55 mg/mL, respectively. These results indicate that *L. casei* TISTR1463 utilized CS-XOS and commercial XOS with similar efficiency. Interestingly, CS-XOS supplementation in MRS medium also enhanced the μ_max_ (0.112 ± 0.010 h^−1^), sugar consumption (2.51 ± 0.04 mg/mL), and lactic acid production (7.65 ± 0.07 mg/mL) of strain TISTR1464 more effectively than commercial XOS, which resulted in values of 0.103 ± 0.003 h^−1^, 2.41 ± 0.10 mg/mL, and 7.03 ± 0.25 mg/mL, respectively. Notably, the μ_max_ of strains TISTR1463, TISTR1464, and TISTR1465 in commercial XOS ranged from 0.100 to 0.101 h^−1^, which were relatively lower than the μ_max_ values observed in CS-XOS treatments (0.110–0.122 h^−1^). These findings demonstrate that CS-XOS effectively promotes the growth of probiotic LAB strains. Therefore, ACSS from CAE (1.0 M NaOH, 90 °C for 30 min), hydrolyzed with recombinant endo-xylanase, was suitable for producing CS-XOS with promising prebiotic activity.

## 4. Discussion

CS primarily contains 12.60–19.10% (*w*/*w*) protein and 16.68–17.70% (*w*/*w*) hemicellulose, making it a promising feedstock for the co-production of bioactive peptides and XOS [27,28,29]. Xylan hemicellulose is typically embedded within the cellulose matrix and shielded by lignin, forming a highly recalcitrant structure. These three major components, cellulose, hemicellulose, and lignin, are interconnected through covalent cross-linkages. Therefore, effective pretreatment of lignocellulosic biomass is essential to disrupt these linkages and enhance the accessibility of target components for downstream enzymatic hydrolysis and bioconversion. The efficiency of MAE relies on the material’s capacity to absorb electromagnetic energy and convert it into heat, thereby facilitating structural disruption of the biomass [29]. Consequently, extending the extraction time can enhance the accessibility of hemicellulose structures, thereby improving XOS recovery yields. Similarly, in the case of UAE, the cavitation effect generated by collapsing bubbles produces localized regions of high temperature and pressure. These effects contribute chemically and mechanically to the disruption of lignocellulosic structures [30]. However, the main drawback of these two techniques lies in the difficulty of scaling up the processes for industrial applications [7].

Alternatively, CAE is simple, cost-effective, and effectively disrupts lignocellulosic structures [31]. This observation is consistent with the findings of Yilmaz Celebioglu et al. (2012) [32], who reported that high concentrations of NaOH could reduce the resistance of CS to chemical breakdown. In addition to breaking down lignin–carbohydrate complexes, alkaline treatment can effectively remove interfering components, such as protein, starch, fat, and soluble sugars, enhancing hemicellulose purity under the same hydrolysis conditions. Based on these observations, 1.0 M NaOH was identified as the optimal concentration for CS pretreatment [32]. However, excessively high alkaline concentrations can lead to the undesirable solubilization of target compounds into the liquid fraction, reducing product recovery. Notably, alkaline treatment also proved highly effective for protein extraction. The concentration of the alkaline solution plays a critical role in protein solubilization. As pH increases, protein extractability improves significantly. For instance, a study reported that increasing the pH of the extraction solvent from 8.0 to 13.0 enhanced rapeseed protein extractability from 33.58% to 61.25% (*w*/*w*), highlighting the positive correlation between alkaline strength and protein recovery [33]. However, a high concentration of NaOH under elevated temperature may induce partial protein hydrolysis, leading to the formation of peptides, as previously reported [34].

Following protein extraction, the recovered protein can serve as a substrate for bioactive peptide production, enhancing its bioactivity and functional value. The most physiologically active peptides typically exhibit low molecular weights and consist of short sequences comprising 2–20 amino acids, generally below 3 kDa in size. This study identified and found three bioactive peptides derived from CS-protein hydrolysate, FLGY, FDYGKY, and FYDTYY, in CS-protein hydrolysate extracted by the MAE method and hydrolyzed by protease_SE5 [7]. Through in silico analysis, these peptides were predicted to exhibit antioxidative, ACE-inhibitory, and DPP-IV inhibitory activities. These tools, such as PepRank, BIOPEP-UWM™, ToxinPred, and SwissADME, are commonly used to assess various peptide-related properties, including physicochemical characteristics, predicted bioactivity, and potential bioavailability. Although these computational tools offer valuable insights for preliminary screening, their outputs should not be interpreted as definitive evidence of biological functionality. Limitations, such as the quality of input data, reliance on existing peptide databases, and constrained model generalizability, should be carefully considered [35,36]. Therefore, experimental validation using *in vitro* and/or *in vivo* assays remains essential to confirm the predicted activities [35].

The in silico predictions of bioactive peptides derived from CS were consistent with the *in vitro* evaluations, particularly for FYDTYY. Other peptides, such as FLGY and FDYGKY, showed partial validation, with approximately 66% consistency between the predicted and experimental results (Table 2). Previous studies have highlighted the critical role of aromatic amino acids, particularly tyrosine (T) and phenylalanine (Y), in antioxidative peptides [17,37]. Their aromatic ring structures may act as electron donors and enhance hydrophobic interactions with cell membranes, thereby facilitating radical scavenging and potentially mitigating cellular inflammation. However, when tested at 200 µg/mL concentrations, the peptides showed no inhibitory effect on LPS-induced nitric oxide (NO) production (Figure 4). This finding aligns with the results of Koh et al. [38], who reported that peptides GY and AF, derived from housefly larvae, exhibited ACE-inhibitory activity via a non-competitive mechanism, which may explain their limited anti-inflammatory potential. These results highlight that bioactive sequences’ position and mode of action are critical factors contributing to the discrepancy between in silico predictions and the actual biological activity observed in cellular systems [39].

A peptide concentration of 200 µg/mL was selected based on preliminary cytotoxicity screening using RAW 264.7 macrophage cells, which confirmed that this dose was non-toxic under the experimental conditions. These findings align with earlier reports demonstrating the low cytotoxicity of short-chain bioactive peptides in macrophage cell models [40,41]. Peptides derived from whey protein hydrolysates containing tyrosine residues have shown no cytotoxic effects in RAW 264.7 macrophages. Notably, the structures of these peptides resemble those examined in this study, as they also contain tyrosine residues at the C-terminal position (GTWY) or within the peptide sequence (DYKKY). Overall, these results support the safety profile of the identified peptides and highlight their potential application as therapeutic agents or functional food ingredients.

Beyond CS-derived bioactive peptides, CS-XOS exhibited prebiotic properties comparable to commercial XOS, underscoring their versatile potential for application across various industries. Similar utilization trends were observed by Gullón et al. [42], who reported the μ_max_ values of 0.04, 0.06, 0.23, and 0.15 h^−1^ for *Lacticaseibacillus casei* L431 cultured in MRS medium supplemented with XOS from barley waste, wheat bran, rice husks, and *Eucalyptus globulus* wood, respectively. Likewise, Boonchuay et al. [19] found μ_max_ values of 0.122, 0.270, and 0.205 h^−1^ for *Lacticaseibacillus casei* TISTR1463, *Lactobacillus delbrueckii* subsp. *lactis* TISTR1464, and *Lactiplantibacillus plantarum* TISTR1465, respectively, when grown on corncob-derived XOS, compared to 0.144, 0.411, and 0.190 h^−1^ with commercial XOS. These results suggest that CS-XOS promotes the growth of probiotic LAB strains at levels comparable to commercial XOS. This supports its potential as a cost-effective prebiotic alternative, particularly considering its origin from agro-industrial by-products. However, further *in vivo* validation is necessary to confirm its functional efficacy.

Notably, the integrated bioprocess developed in this study enabled the co-production of CS-derived bioactive peptides with diverse functional properties and prebiotic CS-XOS. These findings underscore the potential of CS as a valuable substrate for the simultaneous generation of high-value functional ingredients. This integrated approach significantly reduces operational complexity and manufacturing costs by eliminating the need for separate processing lines. Therefore, the proposed strategy offers a more sustainable and economically viable pathway for the valorization of CS, supporting its application as an active ingredient in functional foods and nutraceuticals.

## 5. Conclusions

This study successfully demonstrated the integrated bioprocess for the simultaneous production of bioactive peptides and prebiotic CS-XOS from CS, a by-product of coffee roasting. The CS-protein from CAE (1.0 M NaOH, 90 °C, 30 min) was enzymatically hydrolyzed using protease_SE5, generating low-molecular-weight peptides (0.302 mg/mL), specifically FLGY, FDYGKY, and FYDTYY. These peptides exhibited predicted and *in vitro* antioxidant, ACE-inhibitory, and DPP-IV-inhibitory activities. These peptides were also non-cytotoxic, confirming their safety for potential application in functional foods. After protein extraction, the ACSS was effectively utilized for CS-XOS production via enzymatic hydrolysis with recombinant endo-xylanase. The resulting CS-XOS (52.5 mg/g ACSS) could promote the growth of probiotic LAB, with prebiotic effects comparable to commercial XOS. This co-production strategy may reduce the need for separate processing steps and improve resource utilization in developing food and nutraceutical products. For practical applications in the food industry, further investigations are needed to evaluate the scalability of the process, the stability of the final products, the formulation, and the safety aspects related to their use in human consumption. Addressing these challenges will support the industrial adoption of CS-derived bioactive peptides and XOS as functional food ingredients.

## Figures and Tables

**Table 1:** Protein, peptide concentration, and the antioxidant activity of CS-protein and CS-protein hydrolysate obtained from CAE.

CS-Protein Extraction			CS-Protein Hydrolysis		
Samples	Volume (mL)	Protein Recovery (mg/g CS)	Volume (mL)	Peptide Concentration (mg/mL)	Antioxidant Activity (µmole TE/L)
250 mL Duran bottle	100	83.12 ± 2.66	100	0.323 ± 0.001	1501 ± 21
5 L Stirred-tank bioreactor	3000	80.64 ± 4.82	3000	0.302 ± 0.014	1511 ± 58

Note: The experiments were performed in triplicate (*n* = 3). The results are reported as mean ± SD. The data in each column is not significantly different at *p* ≤ 0.05 according to analysis by *t*-test. ABTS radical scavenging assay was employed to evaluate the antioxidant activity of CS-protein hydrolysate.

**Table 3:** XOS concentration, antioxidant activity, and TPC of CS-XOS from flask and bioreactor scale production.

Production Scale	XOS Concentration (mg/g ACSS)	Antioxidant Activity (µmole TE/ mg XOS)			TPC (mg GAE/mg)
		ABTS	DPPH	FRAP	
250 mL Duran bottle	20.30 ± 0.10	85.01 ± 2.94	36.00 ± 0.75	39.42 ± 3.29	71.67 ± 2.04
5.0 L Stirred-tank bioreactor	52.5 ± 0.08 *	89.43 ± 1.31	32.05 ± 1.24	35.77 ± 2.11	74.56 ± 2.91

Note: The experiments were performed in triplicate (*n* = 3). The results are reported as mean ± SD. The significant differences between CS-XOS production by Duran bottle and stirred-tank bioreactor at *p* ≤ 0.05 are noted by an asterisk (*) according to *t*-test analysis.

**Table 4:** Growth parameters of probiotic LAB cultivated in MRS medium supplemented with commercial XOS and CS-XOS.

Strains	Prebiotics	µ_max_ (h^−1^)	Sugar Consumption (g/L)	Lactic Acid (g/L)
*Lacticaseibacillus casei* TISTR1463	Commercial-XOS	0.103 ± 0.001 ^b^	5.59 ± 0.05 ^d^	9.19 ± 0.55 ^b,c^
	CS-XOS	0.100 ± 0.021 ^b^	5.16 ± 0.17 ^c^	8.49 ± 0.45 ^c^
*Lactobacillus delbrueckii* subsp. *lactis* TISTR1464	Commercial-XOS	0.103 ± 0.003 ^b^	2.41 ± 0.10 ^a^	7.03 ± 0.25 ^a^
	CS-XOS	0.112 ± 0.001 ^ab^	2.51 ± 0.04 ^a^	7.65 ± 0.07 ^b,c^
*Lactiplantibacillus plantarum* TISTR1465	Commercial-XOS	0.104 ± 0.001 ^b^	4.22 ± 0.14 ^b^	7.45 ± 0.26 ^a,b^
	CS-XOS	0.122 ± 0.002 ^a^	4.23 ± 0.17 ^b^	7.47 ± 0.51 ^b,c^

Note: The experiments are performed in triplicate (*n* = 3). Different letters (a–d) within columns are significantly different at *p* ≤ 0.05 according to analysis by Duncan’s multiple range test.

## Data Availability

The original contributions presented in this study are included in the article/Supplementary Material. Further inquiries can be directed to the corresponding author.

## Supplementary Materials

The following supporting information can be downloaded at https://www.mdpi.com/article/10.3390/foods14152745/s1: Table S1. List of identified peptides from CS-protein by LC-MS/MS coupled with *de novo* sequencing. Table S2. List of identified peptides from CS-protein hydrolysate by LC-MS/MS coupled with *de novo* sequencing.
