# Supplementary material for: Valorization of Coffee Silverskin via Integrated Biorefinery for the Production of Bioactive Peptides and Xylooligosaccharides: Functional and Prebiotic Properties
Source: Foods. 2025 Aug 6;14(15):2745. doi: 10.3390/foods14152745 (PMC12345723; doi:10.3390/foods14152745)
Supplement: Supplementary file 1 [file foods-14-02745-s001.zip › foods-3752914-supplementary.pdf]

Supplementary Materials

Table S1. List of identified peptides from CS-protein by LC-MS/MS coupled with *de novo* sequencing.

No.	Peptide	Tag Length	Local Confidence	ALC score	m/z
**F3**					
1	FEGW	4	94 83 82 68	82	538.2277
2	LGFY	4	86 66 92 91	84	499.2536
3	DGSF	4	94 83 82 68	82	425.1689
4	DAPF	4	87 79 75 81	81	449.199
5	YVDF	4	83 75 85 80	81	543.2502
6	HTFEE	5	78 92 95 91 79	88	662.2741
7	CTTLF	5	91 86 85 90 79	86	584.2693
8	KGMYF	5	84 73 75 81 97	82	645.3025
9	HSSPT	5	79 88 94 80 65	82	528.2381
10	LCSEW	5	66 70 90 94 84	81	637.2623
11	YFVVG	5	82 86 95 82 58	81	584.3037
12	ALGDW	5	97 95 87 76 43	80	561.2651
13	YEGFTT	6	93 93 75 85 62 78	81	359.1594
14	YDYDYG	6	93 93 75 85 62 78	81	795.2759
15	TTSCPP	7	98 96 97 94 83 53 41	81	662.2839
16	DAGTFM	7	71 89 66 85 98 59 85	80	770.3047
17	AEGDVA	8	63 91 78 81 86 62 78 97	80	827.3165
18	CVAKAG	9	80 78 77 72 94 58 86 86 81	80	811.3333

Table S2. List of identified peptides from CS-protein hydrolysate by LC-MS/MS coupled with *de novo* sequencing.

No.	Peptide	Tag Length	Local Confidence	ALC score	m/z
**F3**					
1	FLGY	4	97 93 89 93	93	499.2525
2	DVWEGGY	5	88 57 82 92 92	82	496.2067
3	ATSSQ	5	75 80 83 85 76	80	493.2254
4	DVWEGGY	7	72 70 81 78 86 93 98	83	413.1717
5	FDTEHGAF	8	89 97 85 88 86 72 76 86	85	462.2010
6	VFDSACAE	8	72 88 90 93 93 70 36 92	80	421.1729
**F4**					
1	FYDTYY	6	91 90 90 90 90 91	91	436.1788
2	FDYGKY	6	90 97 98 99 95 89	95	396.6815
3	YPAHGTM	7	94 84 95 93 82 97 77	89	388.6761
4	ADKSAEH	7	73 90 80 88 89 97 93	87	379.1776
5	YSSADTV	7	96 92 86 84 87 76 58	83	371.6642
6	PGVCETFH	8	96 95 97 87 41 74 83 98	84	445.1981
7	APSCGPNAP	9	90 92 81 92 86 66 86 94 96	87	407.1797
8	DDDTNSVGH	9	72 96 73 89 88 83 80 75 86	83	480.1888
9	GWATPPCAP	9	75 80 83 92 70 87 89 94 95	85	450.2056
**F5**					
1	KTPKPVK	7	77 93 95 97 79 75 62	83	399.266
